# Incremental value of QT interval for the prediction of obstructive coronary artery disease in patients with chest pain

**DOI:** 10.1038/s41598-021-90133-6

**Published:** 2021-05-18

**Authors:** Dong-Hyuk Cho, Jimi Choi, Mi-Na Kim, Hee-Dong Kim, Soon Jun Hong, Cheol Woong Yu, Hack-Lyoung Kim, Yong Hyun Kim, Jin Oh Na, Hyun-Ju Yoon, Mi-Seung Shin, Myung-A Kim, Kyung-Soon Hong, Wan Joo Shim, Seong-Mi Park

**Affiliations:** 1 Division of Cardiology, Yonsei University Wonju College of Medicine, Wonju, Republic of Korea; 2 Division of Cardiology, Korea University Anam Hospital, Seoul, Republic of Korea; 3 Division of Cardiology, Soon Chun Hyang University Cheonan Hospital, Cheonan, Republic of Korea; 4 Division of Cardiology, Seoul National University Boramae Hospital, Seoul, Republic of Korea; 5 Division of Cardiology, Korea University Ansan Hospital, Ansan, Republic of Korea; 6 Division of Cardiology, Korea University Guro Hospital, Seoul, Republic of Korea; 7 Division of Cardiology, Chonnam National University Hospital, Gwangju, Republic of Korea; 8 Division of Cardiology, Gachon Medical School Gil Medical Center, Incheon, Republic of Korea; 9 Division of Cardiology, Hallym University Chuncheon Sacred Heart Hospital, Chuncheon, Republic of Korea; 10 Department of Cardiology, Korea University Anam Hospital, Korea University College of Medicine, 73, Goryeodae-ro, Seongbuk-gu, Seoul, 02841 Republic of Korea

**Keywords:** Cardiology, Cardiovascular biology

## Abstract

Identification of obstructive coronary artery disease (OCAD) in patients with chest pain is a clinical challenge. The value of corrected QT interval (QTc) for the prediction of OCAD has yet to be established. We consecutively enrolled 1741 patients with suspected angina. The presence of obstructive OCAD was defined as ≥ 50% diameter stenosis by coronary angiography. The pre-test probability was evaluated by combining QTc prolongation with the CAD Consortium clinical score (CAD2) and the updated Diamond-Forrester (UDF) score. OCAD was detected in 661 patients (38.0%). QTc was longer in patients with OCAD compared with those without OCAD (444 ± 34 vs. 429 ± 28 ms, p < 0.001). QTc was increased by the severity of OCAD (P < 0.001). QTc prolongation was associated with OCAD (odds ratio (OR), 2.27; 95% confidence interval (CI), 1.81–2.85). With QTc, the C-statistics increased significantly from 0.68 (95% CI 0.66–0.71) to 0.76 (95% CI 0.74–0.78) in the CAD2 and from 0.64 (95% CI 0.62–0.67) to 0.74 (95% CI 0.72–0.77) in the UDF score, respectively. QT prolongation predicted the presence of OCAD and the QTc improved model performance to predict OCAD compared with CAD2 or UDF scores in patients with suspected angina.

## Introduction

Identification of obstructive coronary artery disease (OCAD) in patients with chest pain is a clinical challenge, particularly in the outpatient clinic. Careful history taking, physical examination and resting electrocardiography (ECG) are important measures in determining initially whether or not to recommend specific tests and invasive coronary angiography (CAG)^1^. Although an abnormal repolarization pattern or presence of Q wave on resting ECG are signs of OCAD, resting ECG should not be used to rule out myocardial ischemia, since it is often normal even in patients with severe OCAD^1^. Updated Diamond-Forrester (UDF) and CAD consortium (CAD2) scores have been used to predict OCAD, based on age, sex, typical symptoms and cardiovascular risk factors, but not ECG findings^2–4^.

Corrected QT (QTc) interval based on resting ECG is a useful marker to stratify patients with increased risk of ventricular arrhythmia^5^. QT prolongation has been reported to predict cardiovascular mortality and sudden cardiac death^5,6^. Various factors including aging, sex, medications and cardiovascular diseases are associated with QT prolongation^7,8^. Myocardial ischemia increased the repolarization heterogeneity of ventricle, and prolonged the duration of electrocardiographic QT interval^9^. In acute coronary syndrome, acute myocardial ischemia was strongly correlated with prolonged QTc interval^10,11^. Although studies have investigated the relation between OCAD and QTc interval^12,13^, it is still unclear whether OCAD is associated with QTc interval in patients with stable angina. We hypothesized that QT prolongation is one of the parameters for the detection of significant OCAD in patients with chest pain.

Therefore, we investigated the association between QTc interval and OCAD. We evaluated the incremental diagnostic value of QT prolongation in conventional tests to identify patients with significant OCAD in clinical practice.

## Results

### Baseline characteristics of population

A total of 1829 consecutive patients visited the outpatient clinic with suspected angina. However, 88 patients were excluded from this study for the following reasons: atrial fibrillation (n = 62); and bundle branch block (n = 26). Finally, our study analyzed 1741 patients (M/F, n = 661/1080; mean age, 61 ± 11 years). Table 1 describes the clinical characteristics of the total study population. Obesity, hypertension, and diabetes mellitus (DM) were observed in 818 (47.0%), 909 (52.2%), and 366 (21.0%) patients, respectively. The mean value of QTc interval was 434.5 ± 31.3 ms. OCAD on CAG was found in 661 patients (38.0%). One-, two-, and three-vessel diseases were diagnosed in 450 (25.8%), 172 (9.9%), and 39 (2.2%) patients, respectively. Stenosis ranging from 50 to 70%, 70% to 90%, and more than 90% was detected in 196 (11.3%), 286 (16.4%), and 179 (10.3%) cases, respectively.

**Table 1:** Baseline characteristics of study population.

	Total	Non-OCAD	OCAD	*p*
	(N = 1741)	(N = 1080)	(N = 661)	
Age, mean (SD)	61.4 (11.3)	59.1 (11.3)	65.3 (10.3)	< 0.001
Men, n (%)	586 (33.7)	336 (31.1)	250 (37.8)	0.004
**Chest pain, n (%)**				< 0.001
Non-specific	248 (14.2)	182 (16.9)	66 (10.0)	
Atypical	640 (36.8)	417 (38.6)	223 (33.7)	
Typical	853 (49.0)	481 (44.5)	372 (56.3)	
Obesity, n (%)	818 (47.0)	502 (46.4)	316 (47.8)	0.361
BMI, mean (SD)	25.0 (3.5)	25.0 (3.5)	24.9 (3.5)	0.573
Hypertension, n (%)	909 (52.2)	501 (46.4)	408 (61.7)	< 0.001
DM, n (%)	366 (21.0)	166 (15.4)	200 (30.3)	< 0.001
Dyslipidemia, n (%)	345 (19.8)	206 (19.1)	139 (21.0)	0.321
Family history of CAD, n (%)	303 (17.4)	180 (16.7)	123 (18.6)	0.166
Current smoker, n (%)	240 (13.8)	123 (11.4)	117 (17.7)	< 0.001
Antidiabetic drug therapy, n (%)	296 (17.0)	132 (12.2)	164 (24.8)	< 0.001
Antihyperlipidemic drug therapy, n (%)	759 (43.6)	421 (39.0)	338 (51.1)	< 0.001
**Antithrombotic drug use**				
Aspirin, n (%)	694 (39.9)	369 (34.2)	325 (49.2)	< 0.001
Clopidogrel, n (%)	418 (24.0)	196 (18.1)	222 (33.6)	< 0.001
**Antihypertensive drug therapy**				
RAS inhibitor, n (%)	549 (31.5)	282 (26.1)	267 (40.4)	< 0.001
CCB, n (%)	482 (27.7)	312 (28.9)	170 (25.7)	0.084
β-blockers, n (%)	385 (22.1)	171 (15.8)	214 (32.4)	< 0.001
Diuretics, n (%)	121 (7.0)	56 (5.2)	65 (9.8)	< 0.001
**Electrocardiography**				
QTc interval (ms)	434.5 (31.3)	429.0 (27.8)	443.5 (34.4)	< 0.001
Heart rate (/min)	70.0 (12.6)	69.1 (91.8)	71.4 (13.8)	< 0.001
QRS duration (ms)	91.3 (28.2)	91.4 (32.6)	91.3 (19.3)	0.932

*OCAD* obstructive coronary artery disease, *BMI* body mass index, *DM* diabetes mellitus, *CAD* coronary artery disease, *RAS* renin angiotensin system, *CCB* calcium channel blockers, *QTc* corrected QT.

In the OCAD, the mean age tended to be older (p < 0.001) and more men were observed compared with non-OCAD (p = 0.004) patients. In the OCAD, the prevalence of hypertension, diabetes mellitus, and current smoking was higher (all p < 0.001). However, obesity and family history of CAD were not different between patients with and without OCAD. Patients with OCAD were treated with a higher number of cardiovascular medications except for calcium channel blockers compared with patients manifesting non-OCAD.

### QTc interval and OCAD

**Figure 1:**
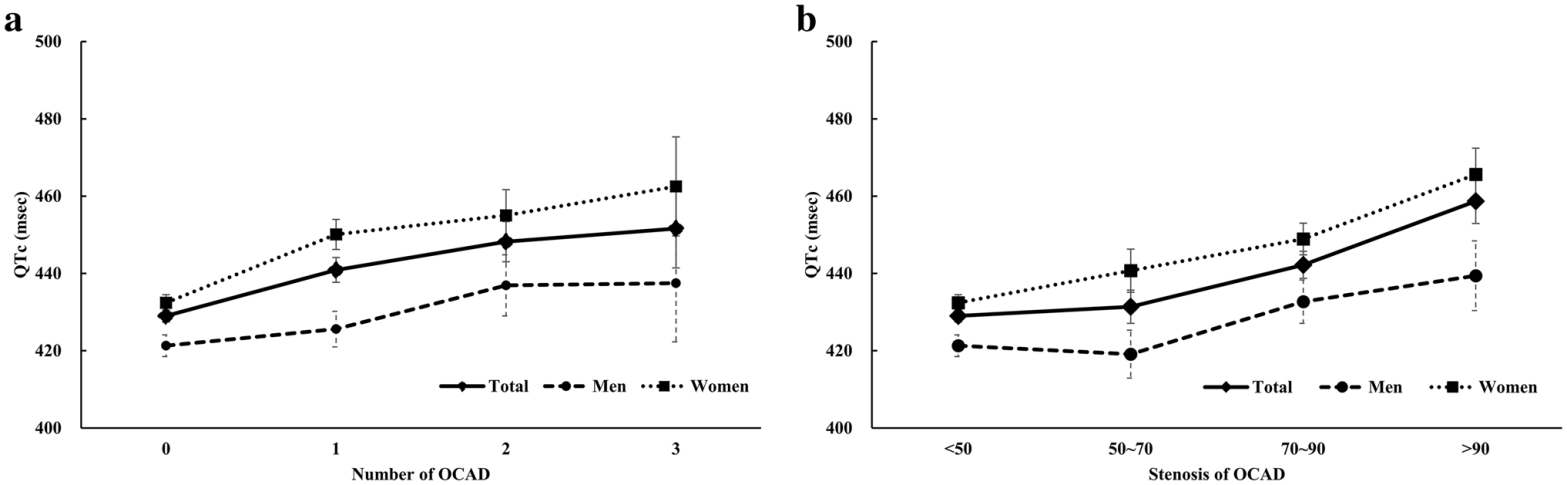
Corrected QT interval according to the number of diseased vessels (**a**) and stenosis (**b**) of OCAD in total subjects, men and women.

There was a significant difference in QTc interval between non-OCAD and OCAD groups (non-OCAD vs. OCAD: 429.0 ± 27.8 vs. 443.5 ± 34.4 ms, p < 0.001). The heart rate on ECG was higher in the OCAD compared with non-OCAD (p < 0.001) patients. The QTc interval was increased according to the degree of stenosis and the number of vessels. The linear trend was statistically significant after adjusting for multiple covariates (all p for linear trend < 0.001) (Fig. 1 and Table 2). The optimal cutoff points of QTc to predict OCAD were 422 and 451 ms in men (AUC, 0.584; confidence interval (CI) 0.543–0.624; P < 0.001) and women (AUC, 0.676; CI 0.648–0.703; P < 0.001), respectively.

**Table 2:** QTc interval according to the severity of obstructive coronary artery disease.

	Total	Men	Women	*p*^†^
	(N = 1741)	(N = 586)	(N = 1155)	
**Number of diseased vessels**				0.004
0	429.0 (27.8)	421.3 (25.4)	432.4 (28.2)	
1	440.9 (34.2)	425.6 (30.2)	450.1 (33.2)	
2	448.2 (34.9)	436.9 (31.9)	455.0 (35.0)	
3	451.6 (31.5)	437.5 (29.6)	462.5 (29.1)	
β (SE), p*	8.1 (1.0), < 0.0001	5.0 (1.5), 0.001	10.0 (1.2), < 0.001	0.010
**Stenosis severity**				0.009
< 50	429.0 (27.8)	421.3 (25.4)	432.4 (28.2)	
50–70	431.4 (30.9)	419.1 (28.6)	440.7 (29.4)	
70–90	442.2 (29.9)	432.7 (30.9)	448.9 (27.4)	
> 90	458.7 (38.9)	439.7 (30.8)	465.6 (39.2)	
β (SE), p*	7.4 (0.7), < 0.001	4.6 (1.18), < 0.001	8.8 (0.8), < 0.001	0.003

Values are presented as mean (SD).

*p-value for trend by using multiple regression model adjusting for age, gender, obesity, diabetes mellitus, hypertension, dyslipidemia, family history of coronary artery disease, current smoking, antihyperlipidemic drug, antidiabetic drug and antihypertensive drug (renin angiotensin system inhibitors, calcium channel blockers, β-blockers, diuretics).

^†^p-value for interaction between gender and number and stenosis of obstructive coronary artery disease.

Table 3 presents the predictive value of QTc interval for OCAD. The additional predictive values of QTc interval were evaluated incorporating in CAD2 and UDF scores. In the CAD2 model, factors such as age, men, typical chest pain, DM and smoking status were independently associated with OCAD. When QTc interval was included in CAD2 model, it was significantly associated with the presence of OCAD (QTc interval; odd ratio (OR), 1.14; CI 1.09–1.18; P < 0.001, QT prolongation; OR, 2.27; CI 1.81–2.85; P < 0.001). In the UDF model, all components including age, sex and typical chest pain were independently associated with OCAD, and the addition of QTc interval was also significantly associated with OCAD (QTc interval; OR, 1.14; CI 1.10–1.19; P < 0.001, QT prolongation; OR, 2.34; CI 1.87–2.93; P < 0.001).

**Table 3:** Predictive value of QTc interval for obstructive coronary artery disease by logistic regression model.

	CAD2 clinical model			
	CAD2 + QTc (continuous)		CAD2 + QT prolongation	
	OR (95% CI)	p-value	OR (95% CI)	p-value
Age (per 10 year)	1.54 (1.38, 1.72)	< 0.001	1.55 (1.39, 1.73)	< 0.001
Male	1.84 (1.43, 2.38)	< 0.001	1.29 (0.997, 1.66)	0.053
**Chest pain**				
Non-specific	1.00		1.00	
Atypical	1.29 (0.90, 1.84)	0.173	1.34 (0.93, 1.92)	0.118
Typical	2.11 (1.46, 3.07)	< 0.001	2.20 (1.51, 3.20)	< 0.001
DM	1.79 (1.38, 2.33)	< 0.001	1.81 (1.39, 2.36)	< 0.001
Hypertension	1.18 (0.94, 1.49)	0.157	1.18 (0.94, 1.49)	0.152
Dyslipidemia	1.16 (0.89, 1.52)	0.277	1.17 (0.89, 1.53)	0.267
Smoking	1.81 (1.30, 2.50)	< 0.001	1.84 (1.33, 2.55)	< 0.001
QTc (continuous)	1.14 (1.09, 1.18)	< 0.001		
QT prolongation			2.27 (1.81, 2.85)	< 0.001

	UDF model			
	UDF + QTc (continuous)		UDF + QT prolongation	
	OR (95% CI)	p-value	OR (95% CI)	p-value
Age (per 10 year)	1.59 (1.43, 1.76)	< 0.001	1.60 (1.44, 1.77)	< 0.001
Male	2.20 (1.73, 2.79)	< 0.001	1.51 (1.19, 1.92)	0.006
**Chest pain**				
Non-specific	1.00		1.00	
Atypical	1.27 (0.89, 1.82)	0.182	1.32 (0.92, 1.89)	0.128
Typical	2.14 (1.52, 3.01)	< 0.001	2.21 (1.57, 3.12)	< 0.001
QTc (continuous)	1.14 (1.10, 1.19)	< 0.001		
QT prolongation			2.34 (1.87, 2.93)	< 0.001

*CAD2* CAD Consortium clinical score, *UDF* updated Diamond-Forrester score, *OR* odds ratio, *CI* confidence interval, *QTc* corrected QT, *DM* diabetes mellitus.

### CAD2 and UDF scores, and new QTc risk score

**Figure 2:**
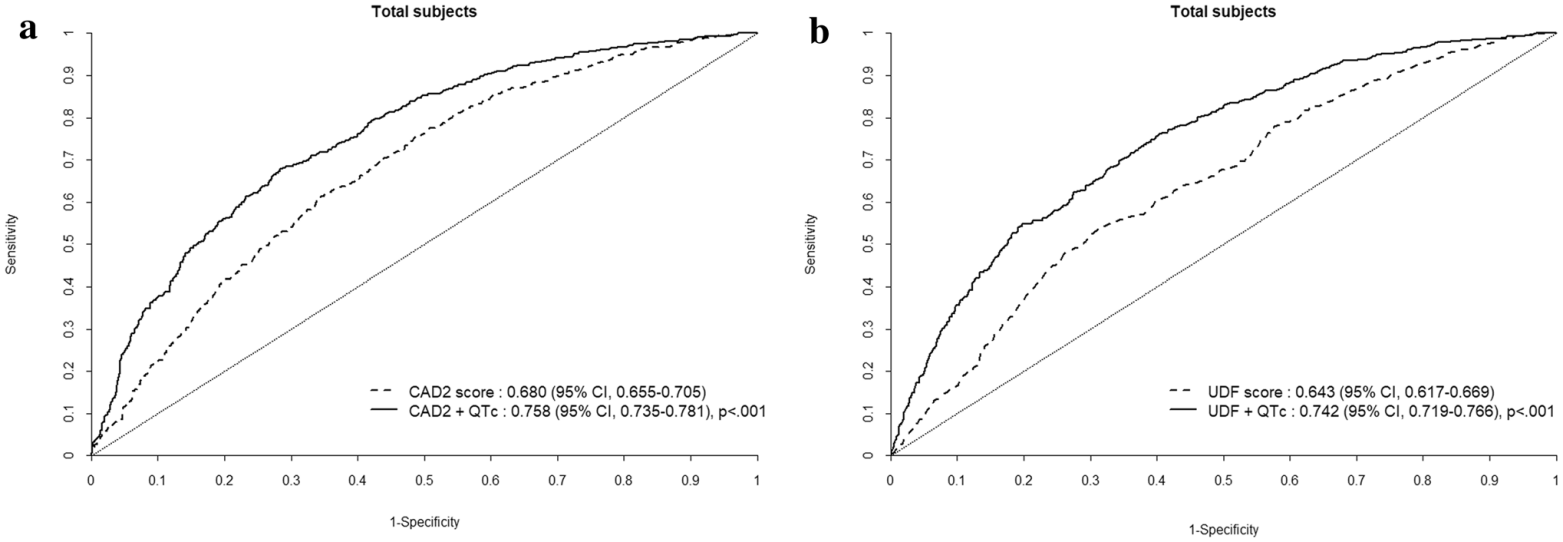
ROC analysis for the additive value of corrected QT interval compared with (**a**) CAD2 score and (**b**) UDF score to predict obstructive coronary artery disease.

Based on continuous ROC analysis, the AUC for CAD2 was 0.680 (CI 0.655–0.705), and for UDF it was 0.643 (CI 0.617–0.669). Figure 2 presents the comparison between CAD2, UDF scores and QTc risk score. After incorporating QTc interval as a component of risk score, the new QTc risk score exhibited significantly higher AUC compared with CAD2 score (0.758, CI 0.738–0.781, p < 0.001). The QTc risk score was also associated with a significantly higher AUC compared with UDF score (0.742, CI 0.719–0.766, p < 0.001).

### Sex differences in the association of QTc interval and OCAD

The QTc interval was longer in women than in men (439.4 ± 31.6 vs. 424.7 ± 11.6 ms, p < 0.001). Occasionally, OCAD on CAG was significantly more prevalent in men than in women (250, 42.7% vs. 411, 35.6%, p < 0.001). In both men and women, QTc was significantly prolonged in patients diagnosed with OCAD compared with those without OCAD (men; 429.3 ± 31.0 vs. 421.3 ± 25.4 ms, p = 0.001, women; 452.1 ± 33.6 vs 432.4 ± 28.2 ms, p < 0.001).

Figure 1 illustrates the association between QTc interval and the severity of OCAD in men and women. In both sexes, QTc interval was increased according to the degree of stenosis and the number of vessels (all p < 0.001), and this linear trend was more prominent in women compared with men (p for interaction; number: 0.014, stenosis: 0.001).

**Figure 3:**
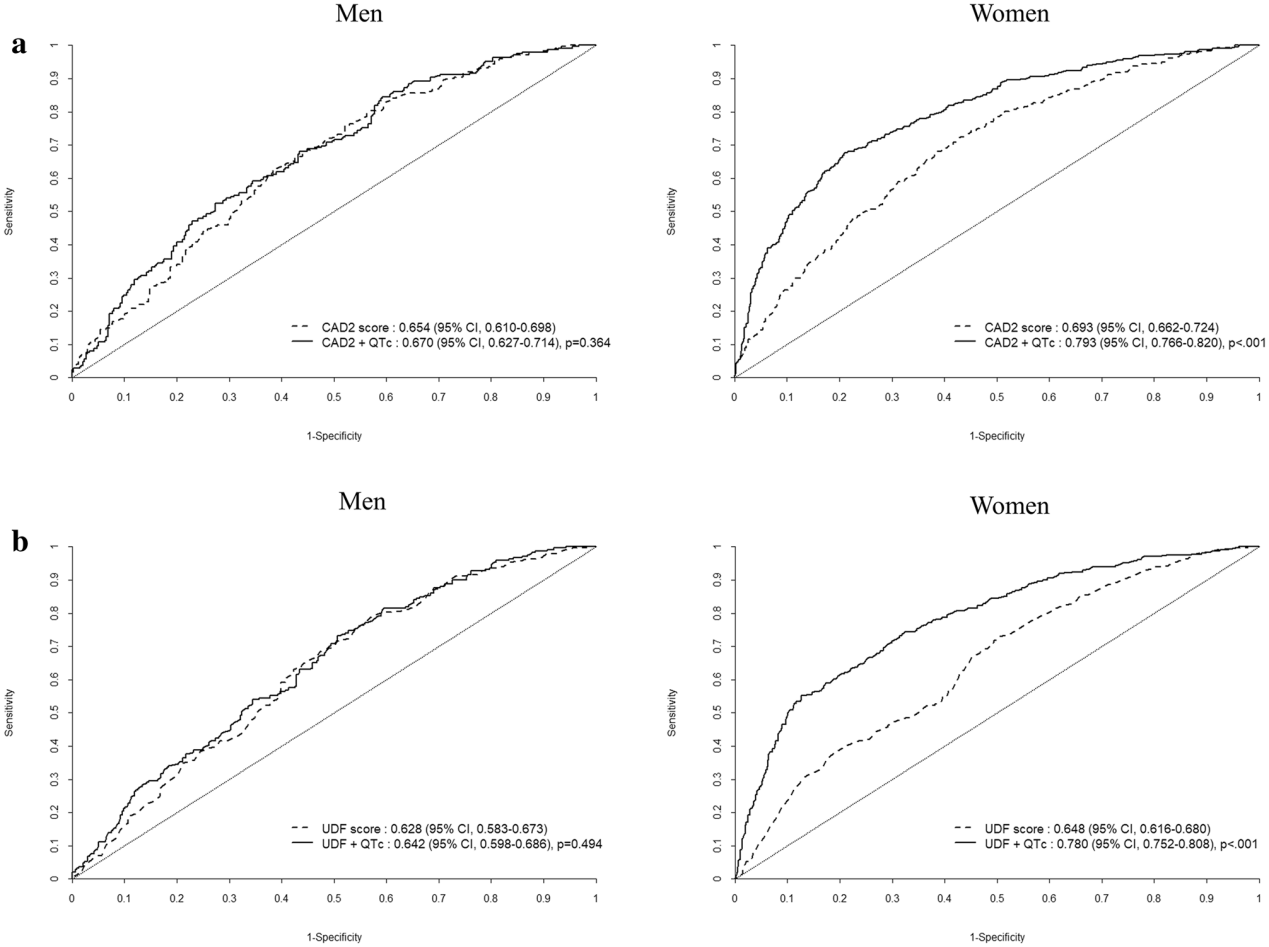
ROC analysis for additive value of corrected QT interval compared with (**a**) CAD2 score and (**b**) UDF score to predict obstructive coronary artery disease in men and women.

Risk scores for the evaluation of pre-test probability of OCAD were compared in men and women. In men, AUC of CAD2 and UDF was 0.654 (CI 0.610–0.698) and 0.628 (CI 0.583–0.673). The AUC of QTc risk score was not significantly different compared with CAD2 or UDF. However, in women, the QTc risk score significantly improved AUC of CAD2 from 0.693 (CI 0.662–0.724) to 0.793 (CI 0.766–0.820) (p < 0.001), and AUC of UDF from 0.648 (CI 0.616–0.680) to 0.780 (CI 0.752–0.808) (p < 0.001) (Fig. 3).

## Discussion

This is the first study demonstrating the predictive value of QT prolongation for the identification of significant OCAD in patients with suspected angina. The study findings were as follows: (1) The mean value of QTc interval was prolonged in patients with OCAD compared with non-OCAD. (2) The QTc interval was increased linearly according to the severity of OCAD, especially in women. (3) Compared with other pretest probability models, the new score, QTc risk score including QTc interval significantly improved the predictability compared with the traditional risk model such as CAD2 or UDF scores.

Physicians frequently encounter patients presenting with chest pain in the outpatient clinic. In these patients, clinical risk profiling is required and numerous diagnostic strategies exist. The previous risk scores were based on age, sex, typical symptoms and cardiovascular risk factors^2–4,14,15^. The rapid risk scores facilitated the identification of high-risk patients; however, many patients were misdiagnosed. Patients misdiagnosed as non-cardiac chest pain constituted nearly a third of cardiovascular deaths and acute coronary syndromes during follow-up^16^. Therefore, it is imperative to develop an accurate and effective predictive model for patients with cardiac chest pain.

In the era of multimodality imaging, the role of anatomical imaging and functional testing has been investigated^17^. Recently, coronary computed tomographic angiography (CCTA) improved the pre-test probability with high accuracy^18^. However, CCTA is associated with radiation hazards, nephrotoxicity due to contrast agents and high costs^1^. ECG is the first, simple, cost-effective and universal “bedside” tool for the evaluation of cardiac chest pain^1^. Among the various parameters of ECG, QTc interval can be easily calculated. Therefore, this study evaluated the incremental predictive value of QTc interval beyond the traditional risk scores.

Acute myocardial ischemia has been reported to increase repolarization heterogeneity of myocardium and prolong the QTc interval^11^. Several possible mechanisms have been suggested. Because sympathetic and neurohormonal systems are associated with QT prolongation^19^, the increased catecholamine levels in ischemic myocardium may result in QT prolongation^6^. Acute myocardial ischemia also alters the myocardial response to catecholamine or cholinergic stimulation, and disturbances associated with potassium or calcium ion channels^9,20^. Beyond the basic mechanisms, several clinical studies reported the association between myocardial ischemia and QTc interval. Nowinski et al. reported that transient myocardial ischemia during balloon inflation in elective coronary angioplasty induced significant changes in ventricular repolarization represented by increased QT interval^21^. It suggests that QTc interval may be an early marker of myocardial ischemia. In 206 consecutive patients reporting to the emergency department for acute chest pain, QTc interval correlated with underlying myocardial ischemia based on functional stress test^10^.

Compared with acute coronary syndrome setting, the role of QTc interval in patients with stable angina has been rarely investigated. In the eighties, Kramer et al. reported the association between the degree of OCAD and QTc interval only in patients with reduced left ventricular function; however, the study, which included a relatively small sample, did not control various factors affecting QTc interval^12^. More recently, Stankovic et al. demonstrated that QTc interval and myocardial contraction duration on echocardiography were related to the presence of OCAD^22^. Our findings about QTc interval and OCAD are consistent with previous studies. The study demonstrates that QTc interval is strongly correlated with the presence and severity of OCAD, suggesting the additional probability of QTc interval to detect significant OCAD in prospective-design, real world study population. QT prolongation also predicted the development of clinical outcomes. The QTc interval before angiography was longer in nonsurvivors than in survivors, and development of QTc prolongation was more frequently observed in patients with sudden death (61%) and acute myocardial infarction (26%) than in those with non-cardiac death (0%)^23^. In patients with chronic ischemic heart disease, QT prolongation was associated with mortality and sudden death^24^.

Although the current study demonstrated the significant impact of OCAD on QTc interval in women, the underlying pathophysiology has yet to be elucidated. Several possible causes have been suggested. First, diastolic disturbances are the earliest mechanical abnormalities associated with ischemia, and abnormal prolongation of the QTc may be a reflection of these changes^25,26^. We previously reported that the severity of CAD was associated with left ventricular diastolic dysfunction, which was more predominant in women^27^. The prominent association between CAD and diastolic dysfunction in women may be one of the mechanisms. Second, increased catecholamine levels and high sympathetic tone are associated with the development of atherosclerosis and left ventricular concentric remodeling^28^. Therefore, enhanced sympathetic tone and autonomic imbalance are closely linked to QT prolongation^6^. Decreased estrogen levels accelerate the progression of autonomic neuropathy in post-menopausal women^29^. Our study demonstrated that the heart rate was much higher in women compared with men, and increased heart rate may be one of the signs underlying increased sympathetic tone, which is linked to QT prolongation in this population with a mean age of about 65 years. An augmented pulsatile load in elderly women may induce pronounced myocardial remodeling, potentially due to sex differences in gene expression of extracellular matrix, and may be the mechanism underlying vulnerable myocardium in women^30^.

This study is a prospective, nation-wide, multicenter and real-world study with adequate sample size. The study utilizes invasive CAG as a gold standard for the evaluation of the severity of OCAD in all patients. It is hard to decide to proceed further diagnostic management including CAG to identify OCAD in patients with chest pain, particularly in the outpatient clinic. Our study provides an insight into the association between QTc interval and OCAD in patients with suspected angina. The simple measurement of QTc interval facilitates the identification and stratification of patients with severe OCAD. Therefore, the role of QTc interval as a therapeutic marker in ischemia management may be investigated in future studies.

Our study has several limitations. First, QTc interval is variable even in same patients depending on medical conditions such as timing of ECG, cardiovascular medications and comorbidities. However, in this study, ECG was performed in all patients during the first visit to outpatient clinic before CAG. Furthermore, after controlling multivariate covariates including cardiovascular medications, the association between the QTc interval and OCAD was significant. Nonetheless, electrolyte imbalance and the use of certain medications, such as quinolones, which may influence the QTc interval, were not investigated. This is another limitation of the current study. The KoROSE study is an on-going prospective study; therefore, long-term data including the changes in QTc interval and newly developed cardiovascular events will be investigated in future studies. Second, the impact of myocardial ischemia in functional stress test on QTc interval has yet to be evaluated. Finally, the number of women was relatively larger than men in the present study, which may attenuate the association between QT prolongation and OCAD in men.

## Conclusion

QT prolongation independently predicted the presence of OCAD and was strongly correlated with the severity of OCAD. The QTc improved model performance to predict OCAD compared with CAD2 or UDF scores in patients with suspected angina and had incremental diagnostic value for the prediction of OCAD as a new risk score.

## Methods

### Subjects

We included 1829 consecutive patients from the Korean Women’s Chest Pain Registry (KoROSE) who underwent invasive CAG. KoROSE is a prospective, nation-wide, multicenter study enrolling patients with suspected angina visiting outpatient clinic. The protocol of this cohort has been previously described^27,31^. Consecutive patients who visited 11 tertiary hospitals were registered from January 2012 to May 2018. Exclusion criteria were as follows: structural heart disease, previous myocardial infarction, or any coronary revascularization, chronic kidney disease on dialysis, malignancy, or inflammatory diseases. For accurate evaluation of QTc interval, patients with atrial fibrillation, bundle branch block and pacemaker were additionally excluded. This study was approved by the institutional review boards of Korea University Anam Hospital, Guro Hospital, Ansan Hospital, Seoul National University Boramae Hospital, Chonnam National University Hospital, Gachon Medical School Gil Medical Center, and Hanllym University Chuncheon Sacred Heart Hospital. Written informed consent was obtained from all study subjects. This study was performed in accordance with the declaration of Helsinki.

Clinical, demographics and anthropometrics parameters besides medical history were recorded. Detailed history including clinical presentation of chest pain and physical examination was evaluated by attending physicians. The typicality of chest pain was evaluated according to the clinical guidelines^1^.

### Electrocardiography

ECG was performed using a standard 12-lead ECG recording at 25 mm/s immediately after physical examination. The QT interval was measured from the beginning of the QRS complex until the end of the T wave. Bazett’s formula was used to correct the QT interval for heart rate (QTc interval = QT interval/√RR interval)^7^. QT prolongation was defined as the best cutoff value indicating OCAD using receiver operating characteristic (ROC) analysis.

### Invasive coronary angiography

Using a standard protocol, a skilled clinician performed invasive CAG via radial or femoral artery. OCAD was defined as ≥ 50% stenosis in a proximal or middle epicardial coronary artery, or a major branch. The stenosis of OCAD was classified as 50–70%, 70–90%, and ≥ 90% and based on the number of vessels involved, it was classified into one-, two-, and three-vessel disease.

### Risk scores to predict obstructive coronary artery disease

In each subject, the risk scores for the prediction of OCAD were calculated. The UDF score consists of age, sex and typicality of chest pain^2^. The CAD2 score additionally includes hypertension, diabetes mellitus, dyslipidemia and smoking status^3^. The new score with QTc prolongation, QTc risk score was calculated using multiple logistic regression analysis of QTc interval along with the components of UDF and CAD2 scores.

### Patient and public involvement

Patients and public were not involved in the design, conduct, reporting or dissemination of this research.

### Statistical analysis

Continuous variables were expressed as mean ± standard deviation and categorical variables were presented as frequencies (percentages). The clinical differences between sexes were compared using the chi-squared or Student's t-test according to the presence of OCAD. To determine the cutoff value of QTc interval for the prediction of OCAD, a ROC curve analysis was used to estimate sensitivity and specificity. The QTc interval cutoff values were determined based on maximum Youden index (J) in men and women. Multivariate logistic regression analysis was used to investigate the impact of QT prolongation on OCAD after adjustment for age, gender, symptom typicality, cardiovascular risk factors and QT prolongation. We also calculated the predictive power of the 3 scores for OCAD according to the area under the curve (AUC) in each score and compared using the Delong test for paired AUC^32^. A P value < 0.05 was considered statically significant. IBM SPSS (Version 24, IBM, NY, USA) and R version 3.0.2 (R development core team, Vienna, Austria) were utilized for statistical analysis.
